# Microglial activation contributes to cognitive impairments in rotenone-induced mouse Parkinson’s disease model

**DOI:** 10.1186/s12974-020-02065-z

**Published:** 2021-01-05

**Authors:** Dongdong Zhang, Sheng Li, Liyan Hou, Lu Jing, Zhengzheng Ruan, Bingjie Peng, Xiaomeng Zhang, Jau-Shyong Hong, Jie Zhao, Qingshan Wang

**Affiliations:** 1 School of Public Health, Dalian Medical University, Dalian, 116044 China; 2 National-Local Joint Engineering Research Center for Drug-Research and Development (R&D) of Neurodegenerative Diseases, Dalian Medical University, No. 9 W. Lvshun South Road, Dalian, 116044 China; 3 Neurobiology Laboratory, National Institute of Environmental Health Sciences, National Institutes of Health, Research Triangle Park, Durham, North Carolina USA

**Keywords:** Neuroinflammation, Apoptosis, Microglial depletion, Cognitive deficits, Parkinson’s disease

## Abstract

**Background:**

Cognitive decline occurs frequently in Parkinson’s disease (PD), which greatly decreases the quality of life of patients. However, the mechanisms remain to be investigated. Neuroinflammation mediated by overactivated microglia is a common pathological feature in multiple neurological disorders, including PD. This study is designed to explore the role of microglia in cognitive deficits by using a rotenone-induced mouse PD model.

**Methods:**

To evaluate the role of microglia in rotenone-induced cognitive deficits, PLX3397, an inhibitor of colony-stimulating factor 1 receptor, and minocycline, a widely used antibiotic, were used to deplete or inactivate microglia, respectively. Cognitive performance of mice among groups was detected by Morris water maze, objective recognition, and passive avoidance tests. Neurodegeneration, synaptic loss, α-synuclein phosphorylation, glial activation, and apoptosis were determined by immunohistochemistry and Western blot or immunofluorescence staining. The gene expression of inflammatory factors and lipid peroxidation were further explored by using RT-PCR and ELISA kits, respectively.

**Results:**

Rotenone dose-dependently induced cognitive deficits in mice by showing decreased performance of rotenone-treated mice in the novel objective recognition, passive avoidance, and Morris water maze compared with that of vehicle controls. Rotenone-induced cognitive decline was associated with neurodegeneration, synaptic loss, and Ser129-phosphorylation of α-synuclein and microglial activation in the hippocampal and cortical regions of mice. A time course experiment revealed that rotenone-induced microglial activation preceded neurodegeneration. Interestingly, microglial depletion by PLX3397 or inactivation by minocycline significantly reduced neuronal damage and α-synuclein pathology as well as improved cognitive performance in rotenone-injected mice. Mechanistically, PLX3397 and minocycline attenuated rotenone-induced astroglial activation and production of cytotoxic factors in mice. Reduced lipid peroxidation was also observed in mice treated with combined PLX3397 or minocycline and rotenonee compared with rotenone alone group. Finally, microglial depletion or inactivation was found to mitigate rotenone-induced neuronal apoptosis.

**Conclusions:**

Taken together, our findings suggested that microglial activation contributes to cognitive impairments in a rotenone-induced mouse PD model via neuroinflammation, oxidative stress, and apoptosis, providing novel insight into the immunopathogensis of cognitive deficits in PD.

**Supplementary Information:**

The online version contains supplementary material available at 10.1186/s12974-020-02065-z.

## Background

Traditionally, Parkinson’s disease (PD) is considered a movement disorder with progressive dopaminergic neurodegeneration and Lewy body formation in the substantia nigra [1]. Current evidence suggests that the pathological processes of PD extend beyond the nigrostratal system and degeneration of neuronal populations in other brain regions has also been noted [2]. Furthermore, patients with PD display not only motor deficits but also a lot of non-motor symptoms [3]. In the clinic, cognitive impairments are frequently observed in PD patients and are often inadequately treated, which gradually becomes an important determinant of life quality of patients [4, 5]. Currently, the mechanisms of cognitive deficits in PD remain unclear.

Microglia-mediated neuroinflammation has been reported to contribute to the pathogenesis of PD [6]. In addition to nigrostriatal regions, activated microglia and accumulation of inflammatory factors are also noted in the hippocampus and cortex, two critical brain regions that may contribute to cognitive decline in PD [7]. Negative correlations between microglial activation and hippocampal volume or cerebral glucose metabolic rate within hippocampus have been observed in PD patients with dementia [8]. Furthermore, Menza et al. found higher levels of proinflammatory factors, such as interleukin-1β (IL-1β) and tumor necrosis factor α (TNFα) in the plasma of PD patients than that of individuals without PD [9]. A subsequent study demonstrated that increased TNFα and TNF receptor 1 contents in the plasma of PD patients are associated with poor cognitive test scores [9]. Similarly, in Alzheimer’s disease (AD) patients, a positive correlation between microglial activation and the clinical dementia rating score has also been observed [10]. Additionally, activated microglia have been shown to induce neurotoxic reactive astroglia [11]. Neurotoxic astroglial activation is also considered to contribute to neuronal damage and subsequent cognitive impairment in animal models of neurodegeneration [12]. Consistently, blocking microglia-mediated conversion of astroglia to a neurotoxic phenotype by IL-10 or fluorocitrate alleviated LPS-induced depressive-like behavior and cognitive dysfunction in mice [13]. These results suggest that neuroinflammation mediated by microglia may contribute to cognitive decline in neurodegenerative disease.

To provide direct experimental evidence linking neuroinflammation and cognitive impairments in PD, a mouse PD model induced by rotenone was administered PLX3397 or minocycline to deplete brain microglia or inhibit microglial activation, respectively. Cognitive performance as well as neuronal loss, synaptic degeneration, and α-synuclein Ser129-phosphorylation were determined. Then, the underlying mechanisms of how microglial activation contributes to cognitive impairments were further explored. Our results suggested that microglial activation damaged cognitive performance in rotenone-induced mouse PD model by exacerbating neuroinflammation, oxidative stress, and neuronal apoptosis.

## Methods

### Reagents

Rotenone was purchased from Sigma-Aldrich, Inc. (R8875, St. Louis, MO, USA). PLX3397 (S7818) and minocycline (S4226) were purchased from Selleck (Shanghai, China). The antibody against Neu-N was purchased from EMD Millipore (MAB377, Temecula, CA, USA). The anti-ionized calcium binding adaptor molecule-1 (Iba-1) and anti-glial fibrillary acidic protein (GFAP) antibodies were purchased from Wako Chemicals (019-19741, Richmond, VA, USA) and Dako (Z0334, Santa Clara, CA, USA), respectively. The antibodies against postsynaptic density protein 95 (PSD-95, ab13552), Ser129 phosphorylated (ab51253), and total α-synuclein (ab6162) were purchased from Abcam (Cambridge, MA). In Situ Cell Death Detection Kit was purchased from KeyGEN BioTECH Corp (KGA7073, Jiangsu, China). The commercial assay kits for glutathione (GSH, S0052) and malondialdehyde (MDA, S1031S) were purchased from Beyotime Biotechnology (Shanghai, China). All other chemicals were of the highest grade commercially available.

### Animal treatment

Eight-week old male C57BL/6 J mice (SPF Animal Laboratory of Dalian Medical University) were randomly assigned to control and two rotenone groups (*n* = 12 in each group). Rotenone was freshly prepared every day in a solution of 0.1% DMSO (diluted with PBS) and was administered daily (i.p., 0.75 or 1.5 mg/kg bw/day) to mice for consecutive 3 weeks as described previously [14, 15]. For the time course study, mice were treated with 1.5 mg/kg bw/day rotenone. After 1, 2, and 3 weeks of initial rotenone injection, mice (*n* = 4 in each time point) were sacrificed and the brains were dissected. Mice in control group were given equal amount of 0.1% DMSO (diluted with PBS). All mice were housed under standard laboratory conditions (a 12-h light/dark cycle with an average room temperature of 25 °C). All animal procedures and their care were performed in strict accordance with the National Institutes of Health guidelines and were approved by the Institutional Animal Care and Use Committee of Dalian Medical University.

### PLX3397 and minocycline treatment

PLX3397 was dissolved in 0.1% DMSO (diluted with PBS) [16]. PLX3397 (40 mg/kg/day) was administered to mice by gavage 7 days prior to rotenone injection (1.5 mg/kg/day, i.p., daily). Based on previous reports and our pilot study, the number of microglia in the PLX3397-treated mice was reduced to approximately 70% of that in control mice at this time point [16, 17]. After 7 days of initial PLX3397 administration, mice (*n* = 12 in each group) received PLX3397 30 mins prior to rotenone by every other day until the end of the experiment. Minocycline (dissolved in PBS, 50 mg/kg/day) was administered (i.p.) to mice 2 days before rotenone (*n* = 11). After 2 days of treatment, minocycline was injected 30 min prior to rotenone by consecutive 3 weeks. The chosen of concentrations of PLX3397 and minocycline was based on previous reports [16, 18]. Mice in control group were given equal amounts of 0.1% DMSO (diluted with PBS).

### Morris water maze test

Morris water maze (MWM) test was performed as described previously [19, 20]. The test included the navigation test and the spatial probe test. In the spatial navigation test, a circular platform was placed in the middle of one quadrant (1 cm below the water surface). In one trial, mice were randomly placed in one of the quadrants and were allowed to swim until they found the platform or for a maximum 90 s. Mice were gently guided onto the platform if they could not find the platform within 90 s, and their latencies were recorded as 90 s. All mice were given a break of 5 min on the platform between trials. There were four trials per day for each mouse, and the mice were tested for 4 days. A video camera was used to record the swimming paths of mice. Different parameters for evaluating learning performance, such as latency for escape and traveled distance were analyzed by using the tracking software (NoldusEtho Vision system, version 5, Everett, WA, USA).

On the fifth day, the spatial probe test for spatial memory function was performed. Briefly, the platform was removed and mice were permitted to navigate in the pool freely for 60 s. Then, different parameters, including the time of mice spent in different quadrants, the latency to initially cross the position where the platform was located and the total number of platform crossings were recorded and analyzed by using the tracking software (NoldusEtho Vision system, version 5).

### Novel objective recognition

Novel objective recognition (NOR) test was conducted based on a previous protocol [21]. In brief, mice were trained over 3 days (3 times per day) to discriminate a novel object from a familiar one. On the first day, mice were put in an empty chamber and permitted to move freely. On the following day, mice were allowed to explore two the same objects placed in opposite corners of the chamber for 5 min. On the last day, mice were put back in the chamber and were permitted to explore a familiar object (the same object as day 2) and a new object with different colors and shapes from the familiar object. After each test, 70% ethanol was used to thoroughly clean the chamber and objects. The time the mice spent exploring familiar and novel objects was recorded, and the recognition index was calculated by using the following formula.

$$ \mathrm{Recognition}\ \mathrm{index}=\frac{\mathrm{Time}\ \mathrm{spent}\ \mathrm{exploring}\ \mathrm{novel}\ \mathrm{object}}{\mathrm{Time}\ \mathrm{spent}\ \mathrm{exploring}\ \mathrm{both}\ \mathrm{novel}\ \mathrm{and}\ \mathrm{familiar}\ \mathrm{object}\mathrm{s}}\times 100\% $$

### Passive avoidance test

The passive avoidance test was done based on previous protocol [22]. In brief, mice were put in a chamber that was separated into light and dark compartments (the same size) by a guillotine door. On the first day, the guillotine door between the light and dark compartments was opened and mice were permitted to move freely for 5 min in both compartments of the chamber. The next day, mice were put in the light compartment. After 60 s, the guillotine door was opened, and mice were permitted to move to the dark compartment freely. Once the mice entered, the guillotine door was closed and an electric shock was given (0.3 mA, 5 min). This test was repeated with 10-min intervals until the latency to enter the dark compartment reached 120 s. On the last day, mice were placed in the light compartment. Sixty seconds later, the guillotine door was opened and the latency of mice to enter the dark compartment (step-through latency) and the number of total entrances (step-through number) were recorded.

### Immunohistochemistry and immunofluorescence staining

Mice in each group were perfused transcardially with PBS, followed by 4% paraformaldehyde and then the brains were collected. Brain samples were postfixed with 4% paraformaldehyde at 4 °C for 48 h, and then transferred to 30% sucrose in PBS before sectioning. Free-floating coronal sections (30 μm) containing hippocampal and cortical regions were used for immunohistochemistry and immunofluorescence staining as described previously [23, 24]. Briefly, brain sections were blocked in 0.25% Triton/PBS containing 4% goat serum for 2 h and then incubated with anti-PSD-95, anti-Neu-N, anti-Iba-1, anti-GFAP or anti-Ser129-phosphorylated α-synuclein antibodies in PBS containing 0.1% Triton X-100 at 4 °C for an additional 24 h. Then, the sections were washed three times prior to incubation with an appropriate biotinylated or Alexa-594-conjugated secondary antibody. Immunohistochemical staining was visualized by using 3,3'-diaminobenzidine. Digital images were acquired (×10 and ×40 magnification for immunohistochemistry and immunofluorescence staining, respectively) under an Olympus microscope (BX51; Olympus, Tokyo, Japan) using an attached digital microscope camera (DP72; Olympus).

The densities of Iba-1, PSD-95, and GFAP immunostaining from two to three brain sections with 120-μm intervals from each mouse in each group were measured by using ImageJ software [24–26]. Briefly, the image was first converted into the grayscale picture, and the background was adjusted before the quantifying area was selected for the measurement of the total pixels. The relative density of the staining was compared based on the density of the total pixels of a certain brain region (total pixels/area). The quantification of the staining was corrected for background staining by subtracting the pixels without primary antibody.

### Automated counting assessment of neurodegeneration

The number of Neu-N^+^ neurons in mice among the groups was quantified by the automated counting method in ImageJ software [19, 24].

### TUNEL assay

In Situ Cell Death Detection Kit was used to perform TUNEL assays in free-floating coronal sections (30 μm) containing hippocampal and cortical regions [22, 27]. TUNEL^+^ cells were observed using a fluorescence microscope (×40 magnification). The mean number of TUNEL^+^ cells in each mouse was obtained by counting three coronal sections with 120 μm intervals by using ImageJ software.

### Western blot analysis

For Western blot analysis, mice were perfused transcardially with PBS to remove the blood. The brains were collected and hippocampal, and the cortical brain regions were dissected immediately on ice. Tissue samples including the hippocampus and cortex were homogenized by using ice-cold RIPA buffer with a protease inhibitor mixture and then centrifuged at 10,000×*g*. After 10 min of centrifugation, protein concentrations were measured in the collected supernatants by using a BCA protein assay kit. Samples containing equal amounts of protein from each group were separated by 4–12% SDS-PAGE, and then, immunoblot analysis was performed using anti-PSD-95, anti-Neu-N, anti-α-synuclein, and anti-α-synuclein (phospho S129) antibodies at 4 °C for 24 h. After washing three times with PBST, the membranes were incubated with appropriate HRP-linked anti-rabbit or mouse IgG. The signal was detected by ECL reagents, and relative density of blots was quantified using ImageJ software.

### Real-time PCR analysis

For RT-PCR analysis, mice in each group (*n* = 6) were perfused transcardially with PBS to remove the blood. The hippocampal and cortical brain regions were dissected immediately on ice and then transferred to liquid nitrogen, followed by a − 80 °C freezer. RNA was isolated from the hippocampal and cortical samples by using TRIzol reagent. Quantitative analysis of RNA was performed by using Nanodrop spectrophotometer. A total 1-μg RNA from each sample was used for complementary DNA (cDNA) synthesis using MuLV reverse transcriptase and oligo dT primers according to previous reports [28, 29]. The reaction conditions were set as 25 °C for 5 min, 42 °C for 60 min, and 70 °C for 15 min. SYBR Premix Ex TaqTM II and a Takara Thermal Cycler Dice™ Real Time System were subsequently used for real-time PCR detection based on the manufacturer’s protocols. The primers were designed with Vector NTI software and validated for efficacy through melting curve analyses. The sequences of the primers were the follows: GAPDH F (5′-TTCAACGGCACAGTCAAGGC-3′; 300 nM), GAPDH R (5′-GACTCCACGACATACTCAGCACC-3′; 300 nM), TNFα F (5′-GACCCTCACACTCAGATCATCTTCT-3′; 300 nM), TNFα R (5′-CCTCCACTTGGTGGTTTGCT-3′; 300 nM), IL-1β F (5′-CTGGTGTGT GACGTTCCCATTA-3′; 300 nM), IL-1β R (5′-CCGACAGCACGAGGCTTT-3′; 300 nM), iNOS F(5′-CTGCCCCCCTGCTCACTC-3′; 300 nM), and iNOS R (5′-TGGGAGGGGTCGTAATGTCC-3′; 300 nM). The following PCR conditions were used: 95 °C for 10 s, 55 °C for 30 s, and 72 °C for 30 s for 40 cycles (for a final reaction volume of 25 μl). All samples were tested in duplicate and normalized to GAPDH using the 2^-ΔΔCt^ method. Fold changes for each treatment were normalized and are shown as percentages of the control.

### GSH and MDA assay

The tissue samples of mice perfused with PBS only were homogenized in ice-cold lysis buffer containing 20 mM Tris (pH 7.5), 150 mM NaCl, 1% Triton X-100, and protease inhibitor mixture. After 10 min of centrifugation at 10,000×*g* (4 °C), the contents of GSH and MDA in the collected supernatant were measured by using commercial kits based on the protocol provided by the manufacturer [30].

### Statistical analysis

Data were expressed as the mean ± SEM. Except for the MWM test, comparison of means among two or more groups was conducted using one-way (one parameter) or two-way ANOVA (two parameters). Subsequently, Tukey’s post hoc test was used for pairwise comparisons between means once ANOVA showed significant differences. The MWM test data were analyzed using repeated measures ANOVA. Fisher’s PLSD tests were used for comparing group means only when a significant *F* value was observed in the overall ANOVA. A *p* value less than 0.05 was considered statistically significant.

## Results

### Rotenone dose-dependently impairs cognitive capacity of mice

**Fig. 1:**
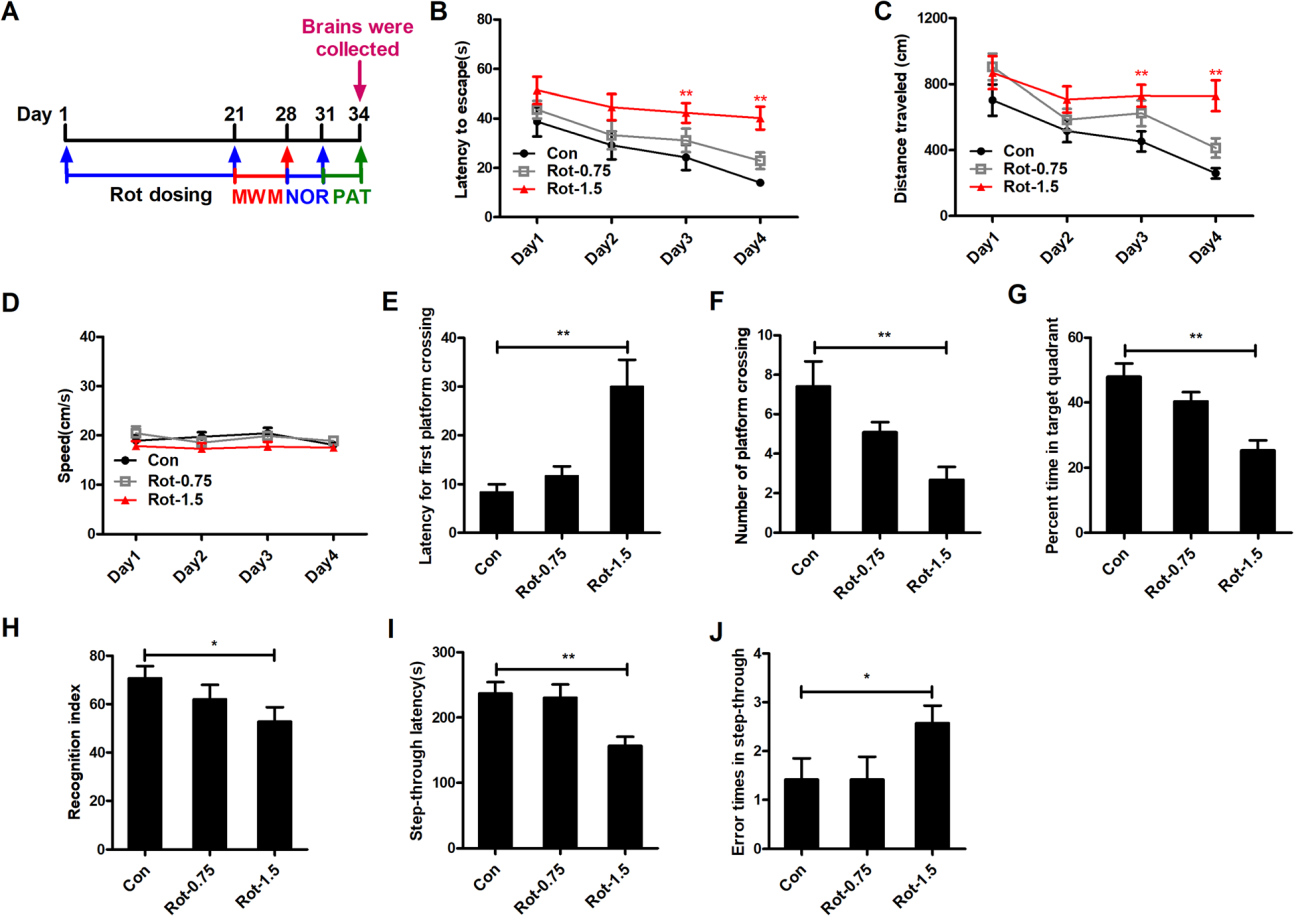
Rotenone dose-dependently impairs the cognitive capacity of mice. **a** Experimental design. Mice were treated with different doses of rotenone. Three weeks after the initial injection, the Morris water maze (MWM) tests were performed, followed by novel objective recognition (NOR) and passive avoidance tests (PAT). After the behavior tests, mice were sacrificed and the brains were collected. For MWM test, different parameters, including escape latency (**b**), distance traveled (**c**), swimming speed (**d**), first platform crossing latency (**e**), number of platform crossings (**f**), and time percentage in target quadrant (**g**) were recorded. **h** For NOR test, recognition index was recorded. For PAT test, step-through latency (**i**) and error times in step-through (**j**) were recorded. *n* = 12; *p < 0.05, **p < 0.01

To determine whether rotenone-treated mice displayed cognitive dysfunction, the MWM, novel objective recognition, and passive avoidance tests were performed after 3 weeks of rotenone exposure (Fig. 1a). In MWZ test, mice in the control group exhibited normal spatial leaning ability as indicated by the time-dependent decreases in escape latency and traveled distance (Fig. 1b). We did not find a significant difference in escape latency and traveled distance between 0.75 mg/kg rotenone-treated mice and vehicle controls, although an increasing trend was observed (Fig. 1b). However, 1.5 mg/kg rotenone significantly elevated the escape latency as the rotenone-treated mice showed a longer time to locate the platform than the control mice (Fig. 1b). Consistently, 1.5 mg/kg rotenone also markedly increased the traveled distance of mice (Fig. 1c), indicating impaired spatial learning function. No significant difference was observed in swimming speed for the mice (Fig. 1d), which excluded the possibility that the differences in MWM performance among the groups were due to locomotor deficits. The spatial probe test was subsequently performed to evaluate the spatial memory capacity of mice. Mice that received 1.5 mg/kg rotenone displayed increased latency for the first platform crossing and a reduced total number of platform crossings and percentage of time spent in the target quadrant compared with control group (Fig. 1e–g), indicating impaired memory ability. However, mice in the 0.75 mg/kg rotenone and control group failed to show significant differences in the spatial probe test (Fig. 1e–g).

To further confirm the impaired cognitive capacity of mice, the novel objective recognition and passive avoidance tests were subsequently performed. Consistently, rotenone at 1.5 mg/kg significantly reduced the recognition index and step-through latency and increased the error times in step-through compared to vehicle controls (Fig. 1h–j). No significant difference in recognition index, step-through latency, and number was observed between 0.75 mg/kg rotenone-treated mice and control group. These results suggest that 1.5 mg/kg rotenone-induced PD model mice displayed impaired cognitive performance.

### Rotenone dose-dependently induces neuronal damage

**Fig. 2:**
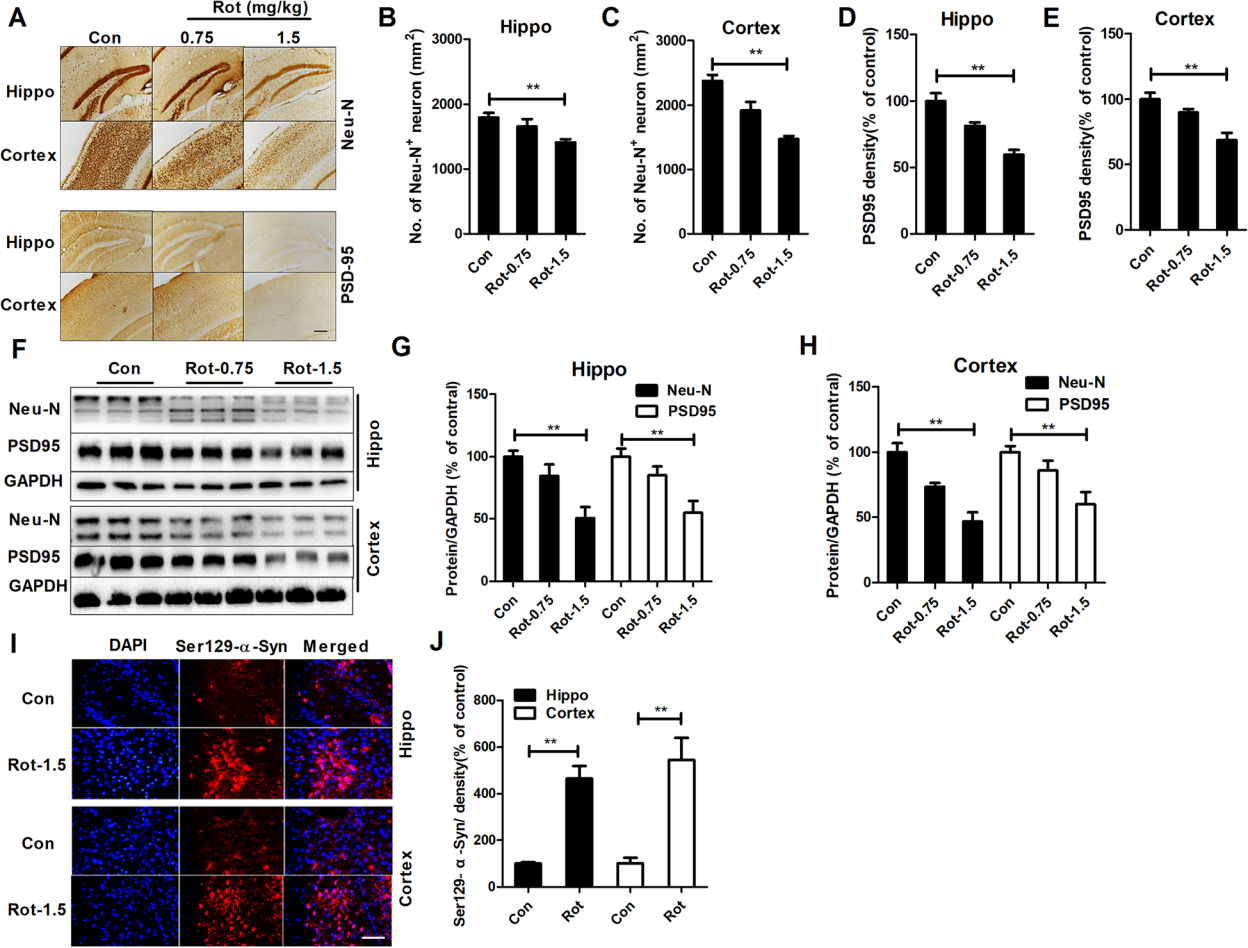
Rotenone dose-dependently induces neurodegeneration and α-synuclein Ser129 phosphorylation in mice. **a** The representative images of Neu-N and PSD95 immunostaining in mice were shown. **b**–**e** Quantitative analysis of Neu-N^+^ cell number and PSD-95 immunostaining density in the dentate gyrus (DG) and cortex of mice. *n* = 5–6. **f** The representative blots of Neu-N and PSD-95 detected by Western blot were shown. **g**, **h** Quantitative analysis of Neu-N and PSD95 blots. *n* = 6. **i** The representative images of Ser129-phosphorylated α-synuclein immunostaining in the hippocampus and cortex of mice were shown. **j** Quantitative analysis of the density of Ser129-phosphorylated α-synuclein immunostaining. *n* = 4. *p < 0.05, **p < 0.01; Scale bar = 200 and 50 μm in A and I, respectively

Cognitive dysfunction in PD is associated with abnormal neuronal damage, especially in the hippocampal and cortical regions [31, 32]. To investigate whether rotenone-induced cognitive deficits were associated with hippocampal and cortical neuron damage, neuronal cells were immunostained with neuron-specific anti-Neu-N antibody. As illustrated in Fig. 2a–e, 1.5 mg/kg rotenone treatment significantly reduced the number of Neu-N+ neurons in the hippocampal and cortical regions of mice compared with the vehicle control treatment. Additionally, synapses are critical for neuron-neuron communication and cognitive function. Reduced expression of key synaptic proteins, such as PSD-95, is significantly correlated with cognitive decline in neurodegenerative disorders [19, 20]. In agreement with neuronal loss, immunohistochemical analysis revealed a reduced density of hippocampal and cortical PSD-95 immunostaining in 1.5 mg/kg rotenone-treated mice (Fig. 2a–e and Supplementary Fig. 1).

To further confirm the damage to neurons in rotenone-treated mice, the levels of Neu-N and PSD95 were determined. As shown in Fig. 2f–h, rotenone at 1.5 mg/kg reduced the protein levels of Neu-N and PSD-95 in the hippocampal and cortical regions of mice. The comparison of Neu-N^+^ cell number, PSD95 immunostaining density and expression of Neu-N and PSD95 between the 0.75 mg/kg rotenone-treated mice and vehicle-treated controls showed no significant differences (Fig. 2a–h).

In addition to neuronal damage, α-synuclein accumulation and phosphorylation, especially at the Ser129 site, also contribute to cognitive deficits in PD [33]. Immunofluorescence staining showed elevated phosphorylation of α-synuclein at Ser129 in the hippocampus and cortex of 1.5 mg/kg rotenone-treated mice compared with control group (Fig. 2i). Fluorescence density analysis of Ser129-phosphorylated α-synuclein immunostaining confirmed this observation (Fig. 2j).

### Microglial activation precedes neuronal damage in rotenone-treated mice

**Fig. 3:**
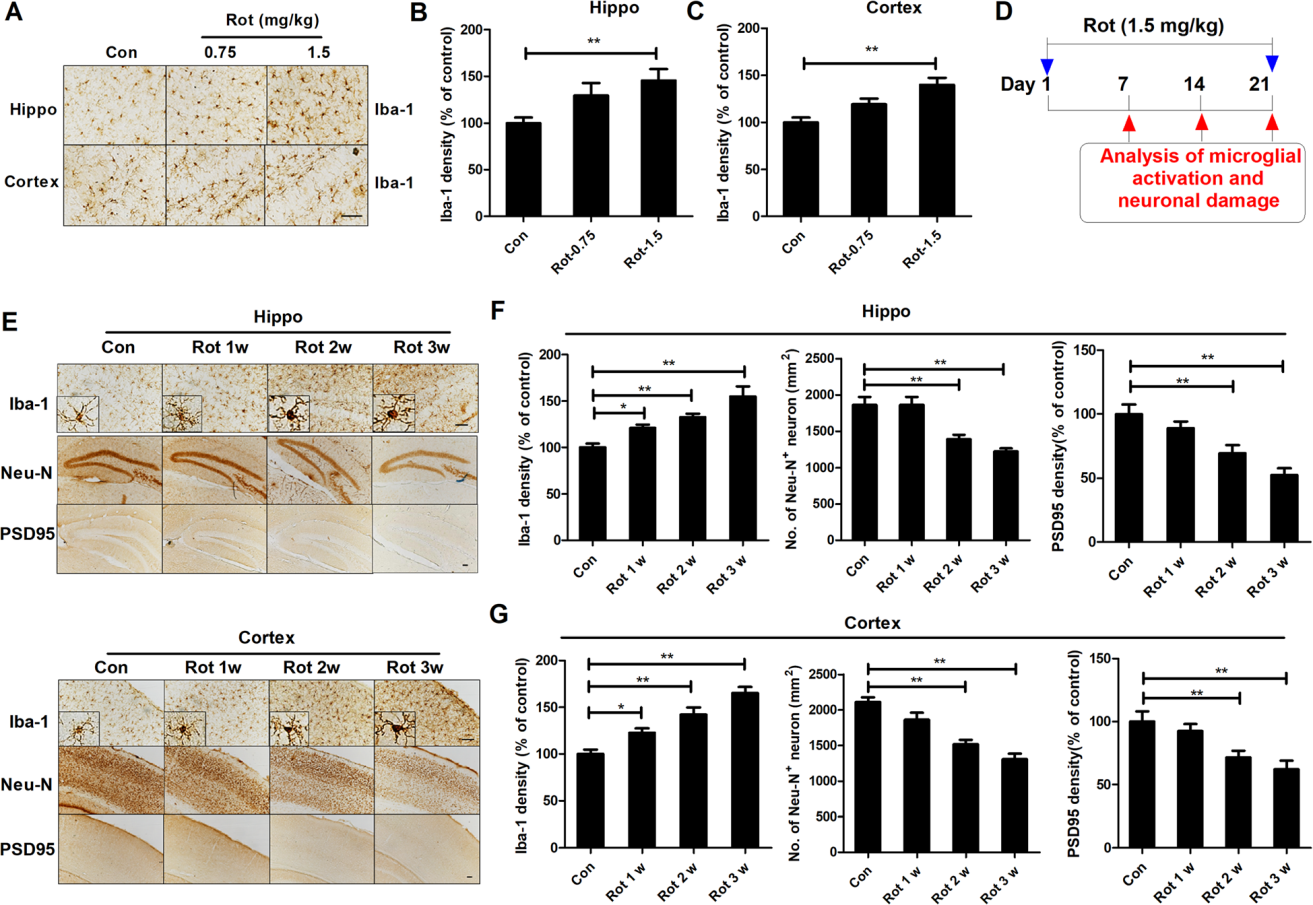
Rotenone-induced microglial activation precedes neurodegeneration in mice. **a** The representative images of Iba-1 immunostaining were shown. **b**, **c** Quantitative analysis of Iba-1 immunostaining density. *n* = 5–6. **d** Experimental design. Mice were administered with 1.5 mg/kg rotenone. After 1, 2, and 3 weeks of initial exposure, Iba-1, Neu-N, and PSD95 were immunostained in the hippocampus and cortex. **e** The representative images of Iba-1, Neu-N, and PSD95 immunostaining were shown. **e**, **f** Quantitative analysis of the densities of Iba-1 and PSD95 immunostaining and the number of Neu-N^+^ cells in mice. *n* = 4. *p < 0.05, **p < 0.01; Scale bar = 50 μm

To investigate whether microglial activation contributes to rotenone-induced cognitive deficits in mice, microglia were immunostained with a microglial marker, Iba-1, after 3 weeks of rotenone exposure. As seen in Fig. 3a, microglial cells in the hippocampus and cortex of 1.5 mg/kg rotenone-injected mice exhibited hypertrophic morphology and increased Iba-1 expression, indicating microglial activation. Quantitative analysis revealed 45.4% and 39.7% increases Iba-1 density in the hippocampus and cortex, respectively, of 1.5 mg/kg rotenone-treated mice compared with controls (Fig. 3b, c). No significant microglial activation was observed in 0.75 mg/kg rotenone-injected mice (Fig. 3a–c).

To determine the time sequence of microglial activation and neuronal damage in rotenone-treated mice, a time course study was performed at the indicated time points of rotenone intoxication (Fig. 3d). The results showed that microglial activation in the hippocampal and cortical regions of mice occurred as early as 1 week after rotenone exposure and was sustained up to 3 weeks (Fig. 3e). Quantitative analysis of Iba-1 immunostaining density revealed 21.1%, 32.6%, and 54.9% increases in the hippocampus and 22.8%, 42.0%, and 65.3% increases in the cortex after 1, 2, and 3 weeks of rotenone injection, respectively (Fig. 3f, g). In contrast, neuronal damage as evidenced by decreased Neu-N^+^ cell number and PSD95 immunostaining density was observed after 2 weeks of rotenone injection (Fig. 3e–g). These results suggested that rotenone-induced microglial activation precedes neurodegeneration.

### PLX3397 and minocycline attenuate rotenone-induced cognitive deficits in mice

**Fig. 4:**
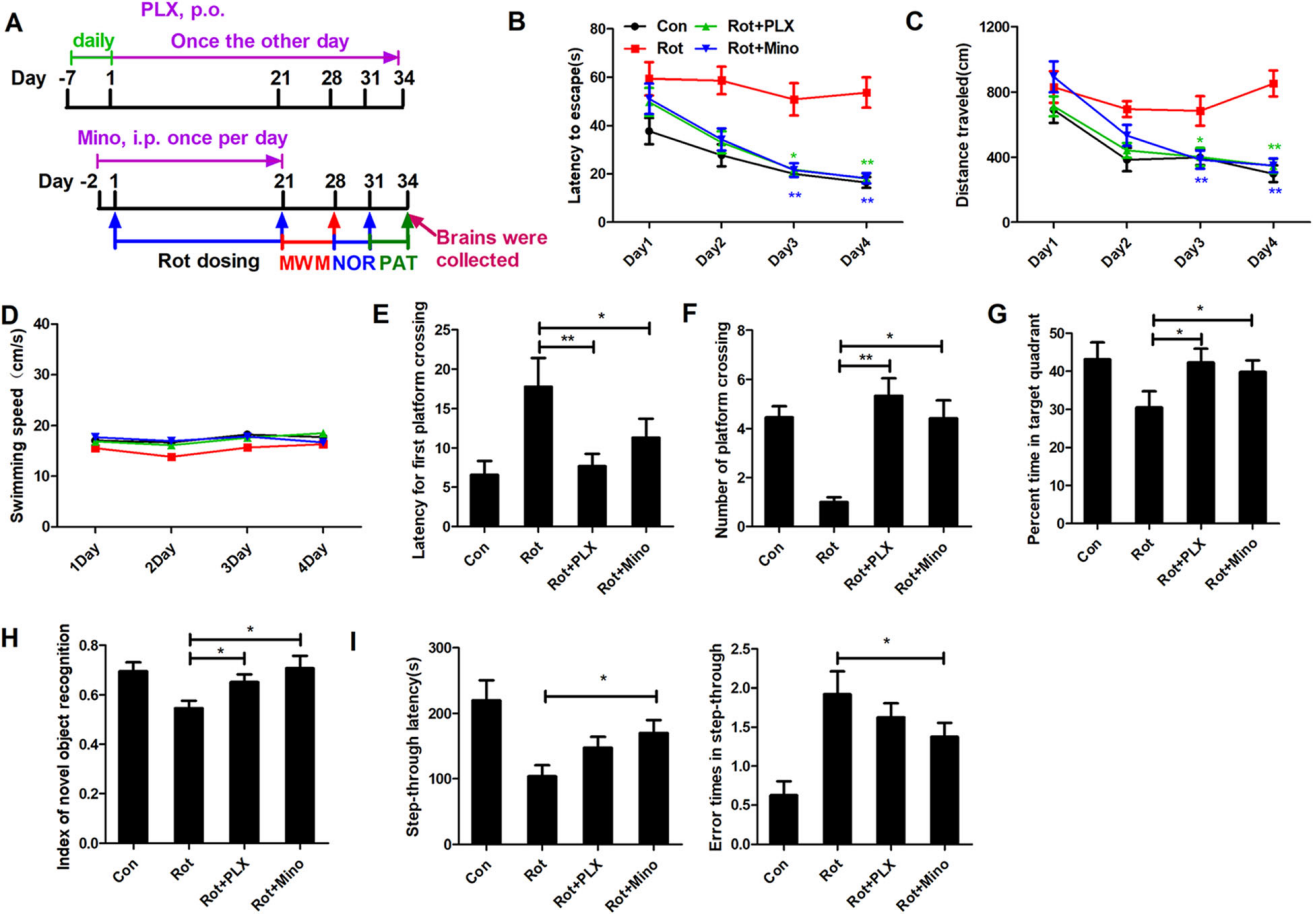
PLX3397 and minocycline attenuate rotenone-induced cognitive deficits in mice. **a** Experimental design. Mice were preadministered with PLX3397 daily. Seven days later, mice received PLX3397 1 h after the rotenone injection every other day until the end of the experiment. Minocycline was administered (i.p.) to mice 2 days before rotenone and continued for consecutive 3 weeks. Morris water maze (MWM) tests were performed and followed by novel objective recognition (NOR) and passive avoidance tests (PAT) in rotenone-treated mice with or without PLX3397 and minocycline. **a** Escape latency, **b** distance traveled, **c** swimming speed, **d** first platform crossing latency, **e** number of platform crossings, and **f** time percentage in target quadrant were recorded. **g**–**i** Novel objective recognition and passive avoidance test were performed in rotenone-treated mice with or without PLX3397 and minocycline. **g** Recognition index, **h** step-through latency, and **i** error times in step-through were recorded. *n* = 11–12. *p < 0.05, **p < 0.01

To determine whether microglial activation contributes to cognitive impairment, rotenone-injected mice were administered PLX3397 or minocycline. PLX3397 is an inhibitor of colony-stimulating factor 1 receptor (CSF1R) and can efficiently reduce the number of microglia in the brains of mice [17]. Minocycline is a widely used antibiotic that could suppress neuroinflammation in a variety of rodent models of neurodegenerative diseases [34]. Consistent with a previous report, PLX3397 treatment significantly reduced the number of microglia in the hippocampus and cortex when compared to vehicle-treated mice (Supplementary Fig 2A-C). Minocycline also efficiently blocked rotenone-induced microglial activation in the hippocampus and cortex of mice (Supplementary Fig 2D-F). Subsequently, cognitive performance was measured in mice treated with rotenone with or without PLX3397 and minocycline. In agreement with the results of Fig. 1, impaired learning and memory performance of mice was detected in MWM test after rotenone (1.5 mg/kg) treatment. Interestingly, PLX3397 and minocycline significantly ameliorated rotenone-induced learning and memory impairments by showing reduced escape latency, traveled distance, and latency for first platform crossing as well as the recovered platform crossing number and percentage of time spent in target quadrant in combined PLX3397 or minocycline and rotenone-treated mice compared with rotenone alone group (Fig. 4a–c and e–g). There were no significant differences in swimming speed among the groups, which excluded the possibility that the protective effects of PLX3397 and minocycline-afforded were caused by recovered motor activity (Fig. 4d).

To further confirm the protective effects of PLX3397 and minocycline against rotenone-induced cognitive deficits, novel objective recognition and passive avoidance tests were performed. In agreement with that of MWZ test, PLX3397, and minocycline significantly elevated the recognition index of rotenone-treated mice in the novel objective recognition test (Fig. 4h). Minocycline also elevated the step-through latency and decreased the error times of step-through in rotenone-treated mice (Fig. 4i). Although reduced error times in step-through and increased step-through latency were observed in PLX3397 and rotenone cotreated mice compared with rotenone alone group, the difference did not reach statistical significance (Fig. 4i). These results suggested that microglial activation contributed to rotenone-induced cognitive deficits in mice.

### PLX3397 and minocycline attenuate rotenone-induced neuronal damage and Ser129 phosphorylation of α-synuclein

**Fig. 5:**
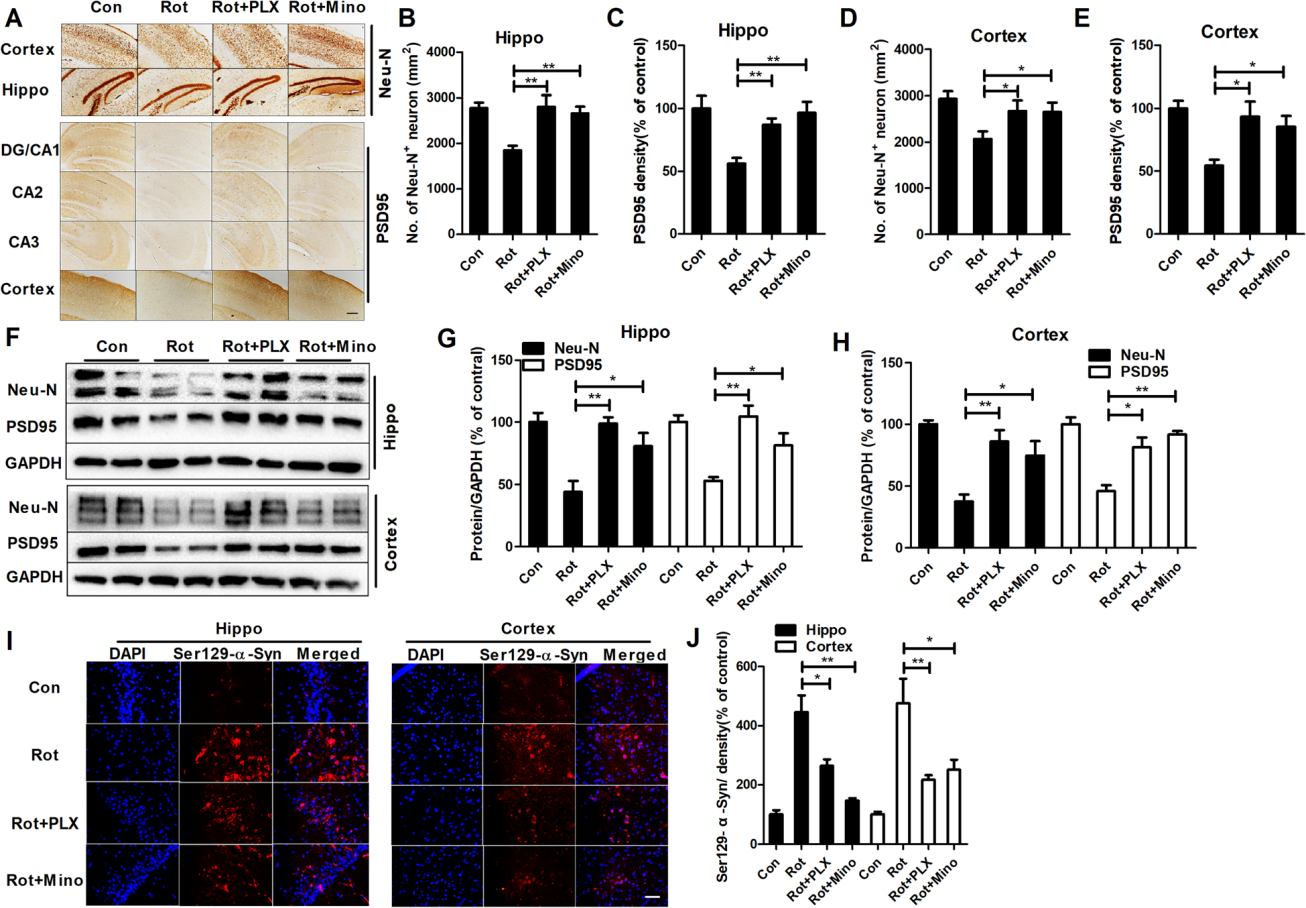
PLX3397 and minocycline attenuate rotenone-induced neuronal damage in mice. **a** The representative images of Neu-N and PSD95 immunostaining in rotenone-treated mice with or without PLX3397 and minocycline were shown. High magnification images of PSD95 staining were shown in Supplementary Fig. 4. **b**–**e** Quantitative analysis of Neu-N^+^ cell number and PSD-95 immunostaining density in the dentate gyrus (DG) and cortex of mice. *n* = 5–6. **f** The representative blots of Neu-N and PSD-95 detected by Western blot were shown. **g**, **h** Quantitative analysis of Neu-N and PSD95 blots. *n* = 4. **i** The representative images of Ser129-phosphorylated α-synuclein immunostaining in the hippocampus and cortex of mice were shown. **j** Quantitative analysis of the density of Ser129-phosphorylated α-synuclein immunostaining. *n* = 4. *p < 0.05, **p < 0.01; Scale bar = 200 and 50 μm in **a** and **i**, respectively

The neuroprotective effects of PLX3397 and minocycline were subsequently determined. Consistent with their attenuation pf cognitive decline, PLX3397 and minocycline also mitigated rotenone-induced neuronal damage in mice. Restored Neu-N^+^ cell numbers and increased PSD95 staining densities were observed in both the hippocampal and cortical regions of PLX3397 or minocycline and rotenone cotreated mice compared with rotenone alone group (Fig. 5a–e and Supplementary Fig. 3), indicating neuroprotection. This conclusion was further supported by analysis of the expression of Neu-N and PSD95 (Fig. 5f–h).

In addition to neuroprotection, PLX3397 and minocycline also mitigated the Ser129-phosphorylation of α-synuclein by showing reduced expression of Ser129-phosphorylated α-synuclein in PLX3397 or minocycline and rotenone cotreated mice compared with rotenone alone group (Fig. 5i, j).

### PLX3397 and minocycline attenuate rotenone-induced astroglial activation and proinflammatory factor production

**Fig. 6:**
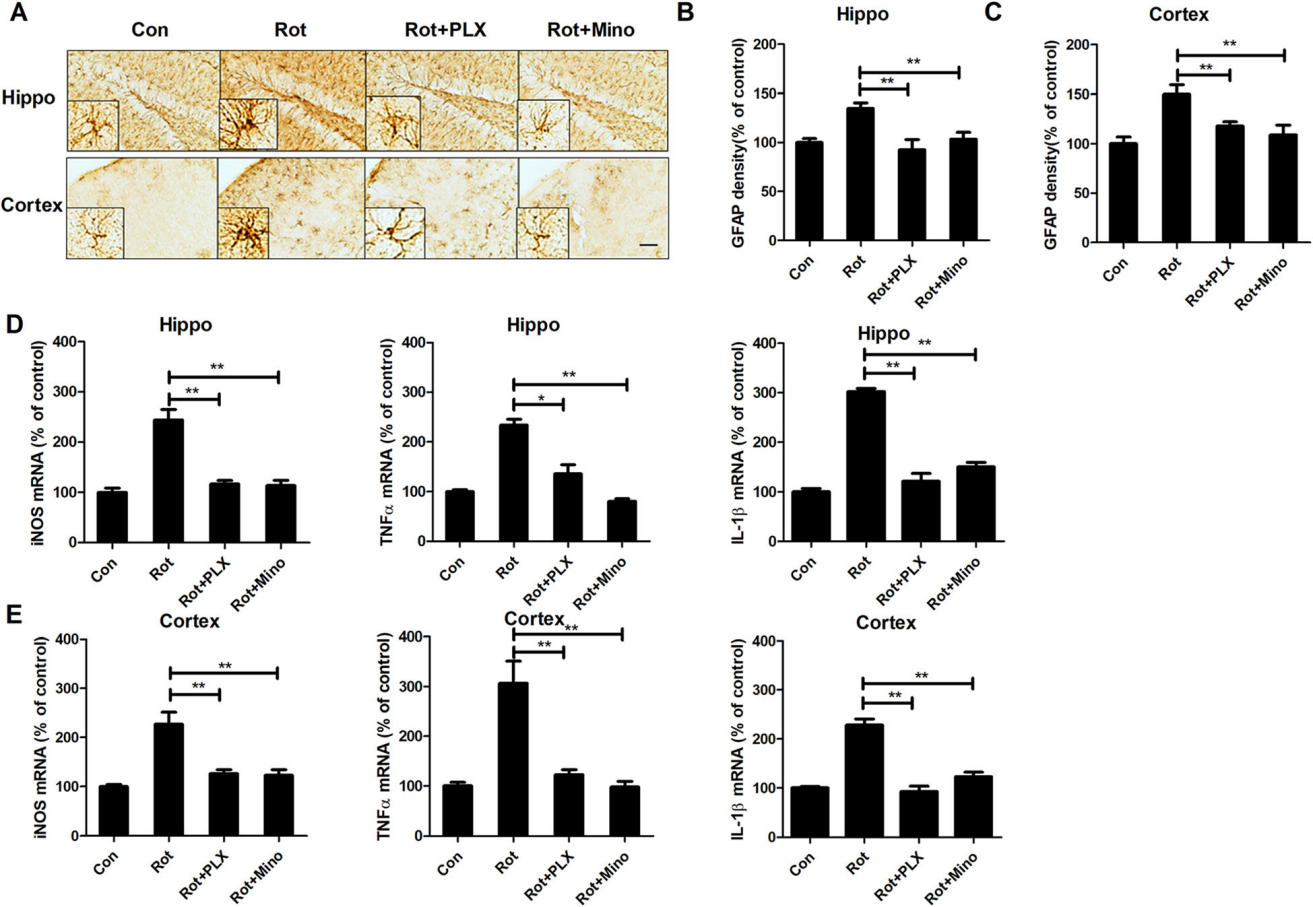
PLX3397 and minocycline attenuate rotenone-induced astroglial activation and gene expression of proinflammatory factors in mice. **a** The representative images of astroglial marker, GFAP immunostaining were shown. **b**, **c** Quantitative analysis of GFAP immunostaining density. **d**, **e** The gene expression levels of iNOS, TNFα, and IL-1β in the hippocampus (**d**) and cortex (**e**) of mice were detected. *n* = 5–6. *p < 0.05, **p < 0.01; Scale bar = 200 μm

A recent study revealed that microglial activation can induce astrocytes into a neurotoxic activation status in multiple neurological disorders [11]. To determine whether microglial depletion by PLX3397 or inactivation by minocycline could suppress astroglial activation, immunohistochemistry using anti-GFAP antibody, was performed in mice. As seen in Fig. 6a, astroglial cells in the hippocampus and cortex of rotenone-treated mice displayed hypertrophied morphology and intensified GFAP immunostaining, which indicated astroglial activation. Densitometric analysis of GFAP staining further supported this conclusion (Fig. 6b, c). Interestingly, astrocytes in PLX3397 or minocycline and rotenone-cotreated mice showed normal morphology and reduced expression of GFAP compared with that of rotenone alone group, suggesting that the activation of astrocytes is blocked by PLX3397 or minocycline (Fig. 6a–c).

The production of proinflammatory factors is a common mechanism by which activated glial cells damage neurons in the central nervous system. To determine whether microglial depletion by PLX3397 or inactivation by minocycline could dampen the production of proinflammatory factors, the gene expression levels of iNOS, TNFα, and IL-1β were measured. As seen in Fig. 6d, e, rotenone injection increased the gene transcript levels of iNOS, TNFα, and IL-1β in both the hippocampus and cortex of mice, and these increases were significantly reduced by PLX3397 and minocycline.

### PLX3397 and minocycline attenuate rotenone-induced oxidative stress

**Fig. 7:**
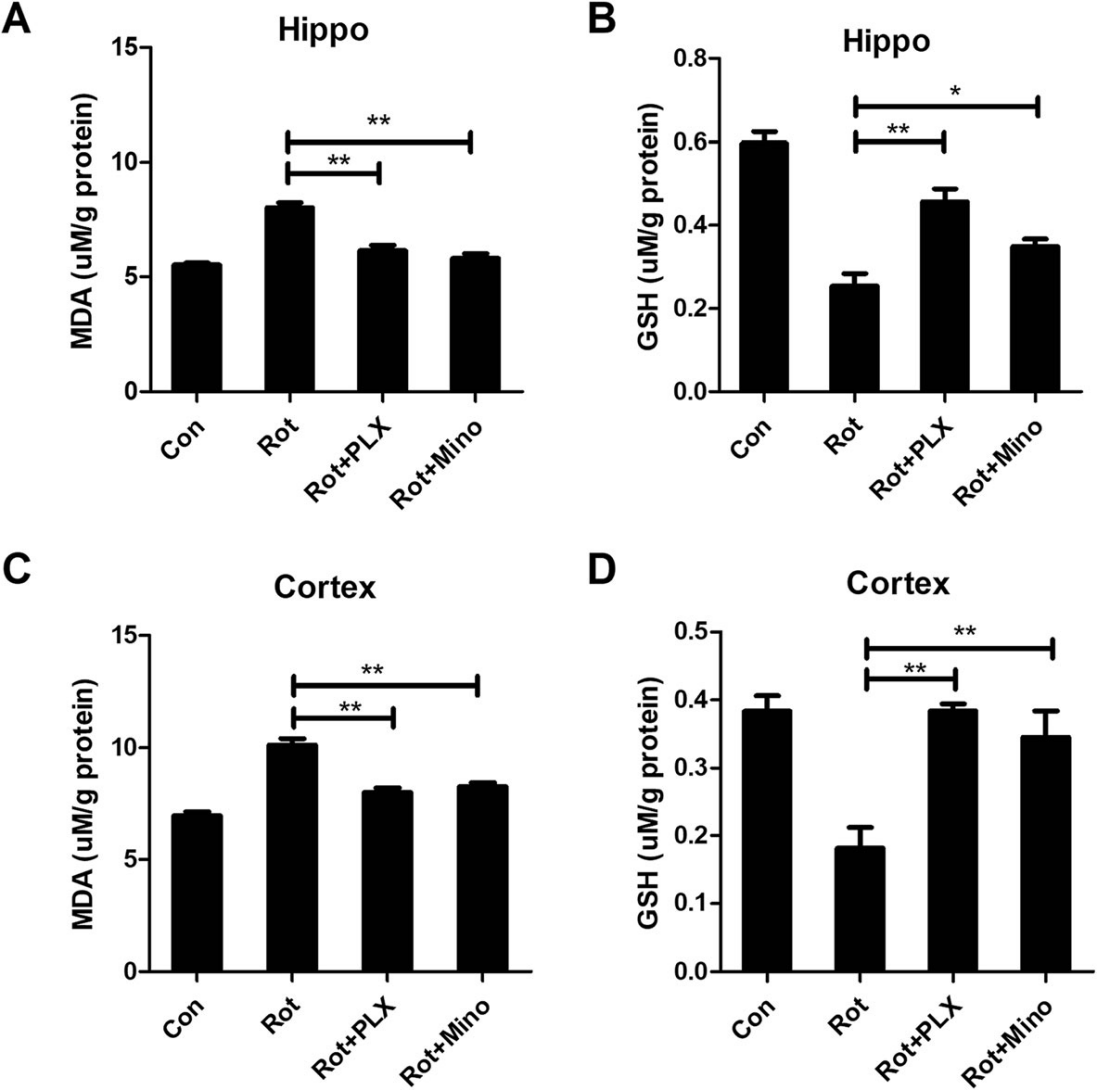
PLX3397 and minocycline attenuate rotenone-induced oxidative stress in mice. The MDA (**a**, **c**) and GSH (**b**, **d**) contents in rotenone-treated mice with or without PLX3397 and minocycline were determined. *n* = 6. *p < 0.05, **p < 0.01

Oxidative stress usually coexists with neuroinflammation. To determine whether microglial depletion by PLX3397 or inactivation by minocycline could dampen oxidative stress, the contents of GSH and MDA were measured. In agreement with reduced production of proinflammatory factors, rotenone-induced lipid peroxidation was also mitigated by PLX3397 and minocycline by showing decreased MDA levels (Fig. 7a, c) and recovered GSH contents (Fig. 7b, d) in PLX3397 or minocycline and rotenone-cotreated mice compared with rotenone alone group.

### PLX3397 and minocycline attenuate rotenone-induced neuronal apoptosis

**Fig. 8:**
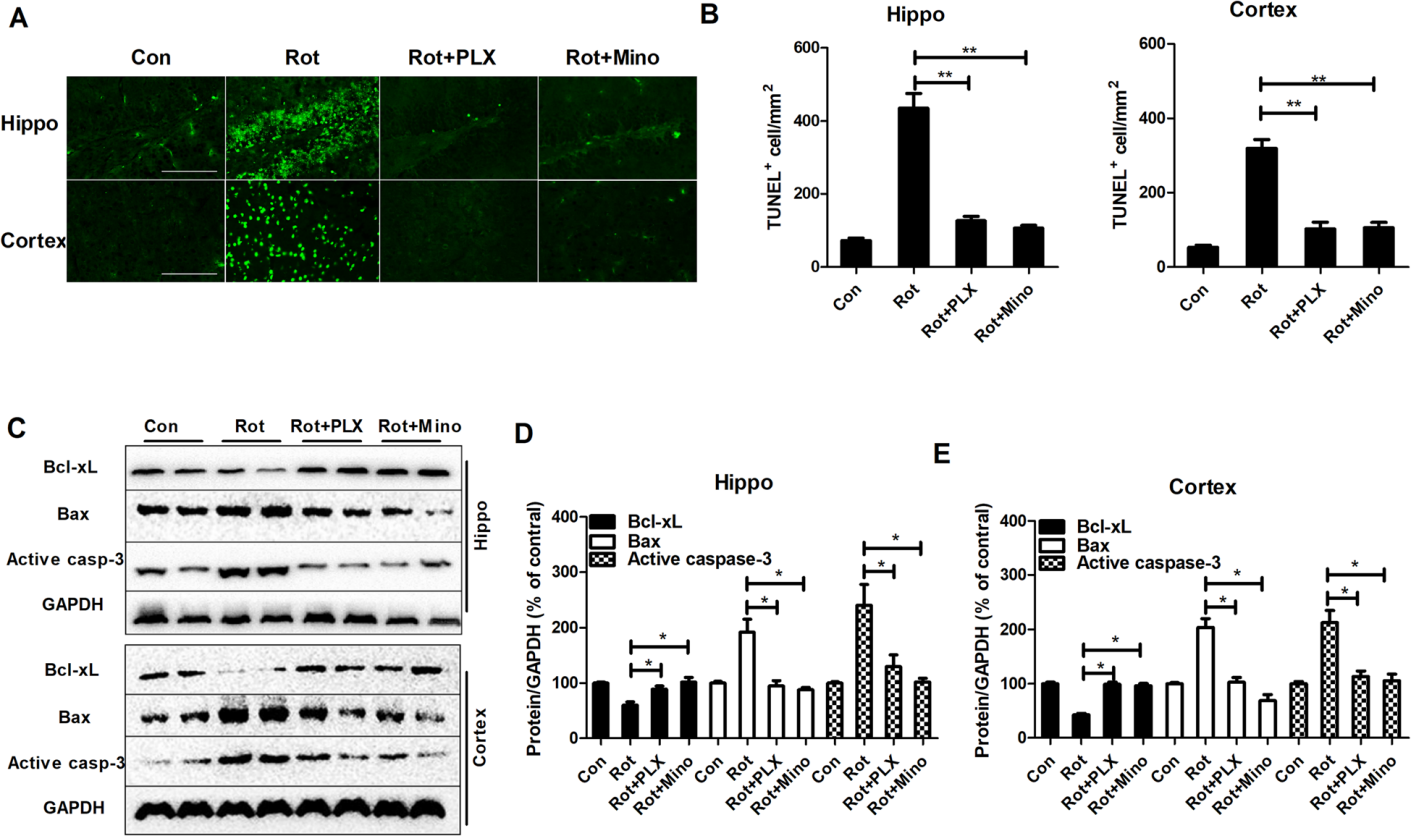
PLX3397 and minocycline attenuate rotenone-induced apoptosis in mice. **a** TUNEL staining was performed in rotenone-treated mice with or without PLX3397 or minocycline. **b** Quantitative analysis of TUNEL^+^ cells. **c** The representative blots of Bcl-XL, Bax, and active caspase-3 detected by Western blot were shown. **d**, **e** Quantitative analysis of Bcl-xL, Bax, and active caspase-3 blots. *n* = 4. *p < 0.05, **p < 0.01; Scale bar = 200 μm

The effects of PLX3397 and minocycline on neuronal apoptosis were further determined in rotenone-treated mice. TUNEL staining revealed a high number of TUNEL^+^ cells in the hippocampal and cortical regions of rotenone-treated mice compared with those of control group (Fig. 8a, b), indicating that apoptosis occurs after rotenone exposure. Immunofluorescence staining with antibodies against MAP2, Iba-1, and GFAP antibodies combined with TUNEL labeling was further performed to observe the distribution of apoptosis among neurons, microglia, and astrocytes, respectively. Results showed that rotenone-induced increase in TUNEL staining in the hippocampus and cortex was mainly concentrated in MAP2^+^ cells, indicating neuronal apoptosis (data not shown). Interestingly, PLX3397 and minocycline treatment markedly decreased rotenone-induced neuronal apoptosis by showing reduced number and percentage of TUNEL^+^ cells in PLX3397 or minocycline and rotenone co-treated mice compared with rotenone alone group (Fig. 8a, b).

It is well documented that apoptosis is controlled by multiple proteins, such as the Bcl-2 family and caspase [35]. Bcl-xL and Bax, two key members of the Bcl-2 family, display anti-apoptotic and proapoptotic functions, respectively [36]. Caspase-3, a member of the caspase family, is the main executor of apoptosis [35]. The effects of PLX3397 and minocycline on the expression of these apoptotic regulators were determined by using Western blotting. Consistent with the increase in TUNEL staining, rotenone treatment caused an increase of active caspase-3 and Bax expression and a decrease in Bcl-xL expression in mice (Fig. 8c–e). In contrast, mice treated with combined PLX3397 or minocycline and rotenone displayed reduced the expression of caspase-3 and Bax as well as elevated expression of Bcl-xL compared with those of mice treated with rotenone alone (Fig. 8c–e), indicating attenuated apoptosis.

## Discussion

In this study, we provided strong evidence to support that microglial activation contributed to cognitive decline in a mouse PD model generated by rotenone. Four salient features were observed: (1) rotenone-induced microglial activation preceded neurodegeneration in mice; (2) microglial depletion by PLX3397 and inactivation by minocycline significantly ameliorated rotenone-induced cognitive deficits and neuronal damage in mice; (3) PLX3397 and minocycline suppressed astroglial activation, production of proinflammatory factors, and oxidative stress in rotenone-treated mice; and (4) PLX3397 and minocycline abrogated rotenone-induced neuronal apoptosis in mice.

PD is traditionally recognized as a movement disorder with progressive loss of nigral dopaminergic neurons [37]. However, accumulating evidence suggests that PD is a heterogeneous multisystem disorder and patients with PD display both motor deficits and multiple nonmotor symptoms [19, 38]. Cognitive impairments are one of the most common nonmotor symptoms in patients with PD. Clinical studies have revealed that 25~46.8% of newly diagnosed patients exhibit mild cognitive deficits, and up to 80% exhibit dementia in the late stage of the disease [39, 40]. A postmortem study in PD patients indicates that limbic and cortical neuron damage and Lewy body pathology correlate with cognitive decline [41]. However, studies in widely used parkinsonian animal model induced by 1-methyl-4-phenyl-1,2,3,6-tetrahydropyridine (MPTP) generated mixed results, which greatly hampered the progress of mechanical studies on cognitive deficits. Although cognitive decline was reported in MPTP-induced mouse model in previous reports [42, 43], Fifel et al. found no significant decrease in cognitive alterations in either acute or chronic MPTP-treated mice [44]. Our preliminary data also showed that MPTP failed to damage learning and memory capacity in mice (data not shown), although significant dopaminergic neurodegeneration was reported in this model [45, 46]. The rotenone-induced mouse PD model is also widely used. However, mixed results regarding the effects of rotenone on cognition in mice have been generated. Alabi et al. reported that daily doses of rotenone (2.5 mg/kg, i.p) for 4 weeks impaired memory in mice as shown by a significantly impaired performance in the Y-maze test compared with that of vehicle controls [47]. In contrast, Jia and colleagues found that intragastric delivery of rotenone (5 mg/kg) for 3 months improved spatial learning and memory abilities of mice in the MWM test, although spatial memory ability was impaired at 1 month after treatment [48]. In this study, we further investigated the effects of rotenone on cognition in mice by using a subchronic dosing regimen. Results showed that rotenone dose-dependently decreased novel objective recognition, passive avoidance, and MWM performance in mice after 3 weeks of treatment. Furthermore, elevated neuronal damage, loss of synapses, and Ser129-phosphorylation of α-synuclein were also detected in rotenone-injected mice, which was consistent with observations in PD patients.

The mechanisms underlying rotenone-induced cognitive dysfunction remain unclear. Inflammation has long been found to be inversely correlated cognitive decline [20, 49]. Increasing evidence suggested that activated microglia is a key causative factor in inflammation-mediated neurodegeneration and behavioral deficits [50]. Dysregulated microglial activation is reported to be able to increase pathological protein aggregation and impair synaptic pruning and neuron plasticity in key brain regions subserving cognition [51, 52]. In the present study, activated microglia were detected in the hippocampus and cortex of rotenone-induced mouse PD model. A time experiment revealed that microglial activation induced by rotenone preceded neuronal damage and α-synuclein pathology in mice. Furthermore, microglial depletion by PLX3397 or inactivation by minocycline significantly reduced neuronal damage in the hippocampus and cortex of mice treated with rotenone. Consistently, neuroprotective effects of both PLX3397 and minocycline against rotenone-induced dopaminergic neurodegeneration were also observed in mice (data not shown). More importantly, PLX3397 and minocycline-afforded neuroprotection were associated with improved cognitive performance in rotenone-treated mice, indicating an important role of activated microglia in mediating cognitive dysfunction. In agreement with our findings, Cope and colleagues recently found that microglial activation plays an active role in obesity-associated cognitive decline since blocking microglial activation by minocycline prevented loss of dendritic spines and cognitive decline in obese mice [53]. Depletion of microglia by CSF1R inhibitors, such as PLX3397 and PLX5622, was also associated with improved cognitive performance in experimental mouse models of AD [54], intracerebral hemorrhage [55], and irradiation-induced memory deficits [56].

Microglia can become overactivated in response to certain injuries and release a variety of cytotoxic factors that cause neurotoxicity [11]. In addition, activated microglia have recently been shown to be able to induce astrocytes into a neurotoxic A1 status by releasing TNFα, IL-1α, and C1q to amplify neuronal damage [11]. Proinflammatory factors and reactive oxygen species (ROS) are considered to be critical to mediate neuroinflammatory damage since neutralization of proinflammatory factors or inhibition of ROS during neuroinflammation showed potent neuroprotection in a variety of neurodegeneration models [57]. Consistent with these findings, in this study, elevated astroglial activation, production of proinflammatory cytokines, and oxidative stress were observed in rotenone-treated mice. Furthermore, we demonstrated that microglial depletion by PLX3397 and inactivation by minocycline suppressed these toxic events and was accompanied by decreased apoptosis of neurons and expression of cleaved caspase-3 and Bax as well as elevated expression of Bcl-xL. Our findings suggested that microglial activation contributed to cognitive deficits and neuronal apoptosis through neurotoxic astroglial activation, neuroinflammation, and oxidative damage. Consistent with our findings, endotoxin LPS, an inflammatory stimulator, has been reported to induce cognitive deficits in mice through neuroinflammation and neuronal apoptosis [21]. Inhibition of inflammatory cytokines, oxidative stress, and neuronal apoptosis in the hippocampus also ameliorated cognitive deficits in mouse model of AD [58], vascular dementia, and sepsis [59]. Notably, a pharmacological manipulation method (PLX3397 and minocycline) was used to block microglial activation in the current study, and we could not exclude the possibility that PLX3397 and minocycline might be have direct effects on astroglial activation, oxidative stress, or neuronal apoptosis. Further study focusing on this issue should be performed in the future.

It is interesting to compare the similar protective effects of minocycline and PLX3397 in this study. Minocycline is a semisynthetic tetracycline derivative that displays potent anti-inflammatory effects. Multiple studies have revealed that minocycline can dampen proinflammatory microglial activation (M1) and might simultaneously promote microglial M2 polarization in neuropathological conditions [60–62]. In contrast, PLX3397 is reported to directly deplete microglia in an apoptosis-dependent manner in the brains of mice [17]. Although microglial elimination by PLX3397 is not accompanied by an inflammatory response in the brain, PLX3397-treated mice exhibited robust reductions in the expression of many inflammatory genes, including TNF-α and other cytokines, in response to LPS [17]. Consistently, Liang et al. reported that PLX3397 treatment significantly reduced the number of M1 phenotype-like microglia and production of proinflammatory factors in a mouse model of high-fat-diet (HFD)-induced obesity [63]. Our preliminary data showed reduced expression of iNOS, a marker of M1 microglia, and elevated expression of the M2 microglial marker Arg-1 in the brains of mice treated with combined PLX3397 and rotenone compared with those of mice treated with rotenone alone (Supplementary Fig. 5), suggesting that PLX3397 might prefer to target/eliminate M1-trended microglia in neuroinflammatory or pathological conditions. Thus, we believe that the equal potency of PLX3397 and minocycline in dampening M1 microglial activation by microglial depletion and inhibition, respectively, might be one of the potential reasons for similar neuroprotective effects of these two compounds.

## Conclusions

Our results reveal an important role of microglial activation in rotenone-induced cognitive deficits in mice through astroglial activation, neuroinflammation, oxidative stress, and apoptosis. Our study adds to strong experimental evidence for the connection between microglial activation and cognitive decline in PD and provides a new therapeutic target for the treatment of the nonmotor symptoms in patients in the future.

## Supplementary Information

**Additional file 1 Supplementary Fig 1.** Rotenone dose-dependently reduces the expression of PSD-95 in CA1, CA2 and CA3 regions of mice. Quantitative analysis of PSD95 immunostaining density in CA1 (A), CA2 (B) and CA3 (C) regions of mice. ** p < 0.01. **Supplementary Fig 2.** The effects of PLX3397 and minocycline on microglial number and activation, respectively. (A) Mice were administered with PLX3397 (40 mg/kg/day) by gavage for 1 week and the representative images of Iba-1 immunostaining were shown. (B, C) Quantitative analysis of Iba-1^+^ cell number. n = 3. (D) The representative images of Iba-1 immunostaining in rotenone-treated mice with or without minocycline were shown. (E, F) Quantitative analysis of Iba-1 immunostaining density. n = 5. * * p < 0.01; Scale bar= 50 μm. **Supplementary Fig 3.** PLX3397 and minocycline attenuate rotenone-induced reduction of PSD95 in CA1, CA2 and CA3 regions of mice. Quantitative analysis of PSD95 immunostaining density in CA1 (A), CA2 (B) and CA3 (C) regions of mice. ** p < 0.01. **Supplementary Fig 4.** PLX3397 and minocycline attenuate rotenone-induced neuronal damage in mice. The representative images (40×) of PSD95 immunostaining in rotenone-treated mice with or without PLX3397 and minocycline were shown. n=5-6; Scale bar= 100 μm. **Supplementary Fig 5.** The effects of PLX3397 and minocycline on expession of iNOS and Arg-1 in the midbrain of rotenone-treated mice. (A) The expression levels of iNOS and arginase-1 (Arg-1) were detected in the midbrain of mice treated with rotenone with or without PLX3397 and minocycline by using Western blot and the representative blots were shown. (B) The blots was quantified. n=4; *p < 0.05, **p < 0.01.

## Data Availability

All data generated or analyzed during this study are included in this published article [and its supplementary information files].

## Abbreviations

AD: Alzheimer’s disease
CSF1R: Colony-stimulating factor 1 receptor
GSH: Glutathione
IL-1β: Interleukin-1β
MDA: Malondialdehyde
MWM: Morris water maze
MPTP: 1-Methyl-4-phenyl-1,2,3,6-tetrahydropyridine
NOR: Novel objective recognition
PD: Parkinson’s disease
PSD-95: Postsynaptic density protein
ROS: Reactive oxygen species
TNFα: Tumor necrosis factor α
