# Correction: Microglial activation contributes to cognitive impairments in rotenone-induced mouse Parkinson’s disease model

**DOI:** 10.1186/s12974-025-03458-8

**Published:** 2025-05-24

**Authors:** Dongdong Zhang, Sheng Li, Liyan Hou, Lu Jing, Zhengzheng Ruan, Bingjie Peng, Xiaomeng Zhang, Jau-Shyong Hong, Jie Zhao, Qingshan Wang

**Affiliations:** 1 School of Public Health, Dalian Medical University, Dalian, 116044 China; 2 National-Local Joint Engineering Research Center for Drug-Research and Development (R&D) of Neurodegenerative Diseases, Dalian Medical University, No. 9 W. Lvshun South Road, Dalian, 116044 China; 3 Neurobiology Laboratory, National Institute of Environmental Health Sciences, National Institutes of Health, Research Triangle Park, Durham, North Carolina USA

**Correction: Journal of Neuroinflammation (2021) 18:4**

10.1186/s12974-020-02065-z

In this article [1], there was an error in Fig. 6A. For completeness and transparency, the old incorrect Fig. 6 and the correct Fig. 6 are displayed below.

**Incorrect Fig.** 6

**Fig. 6:**
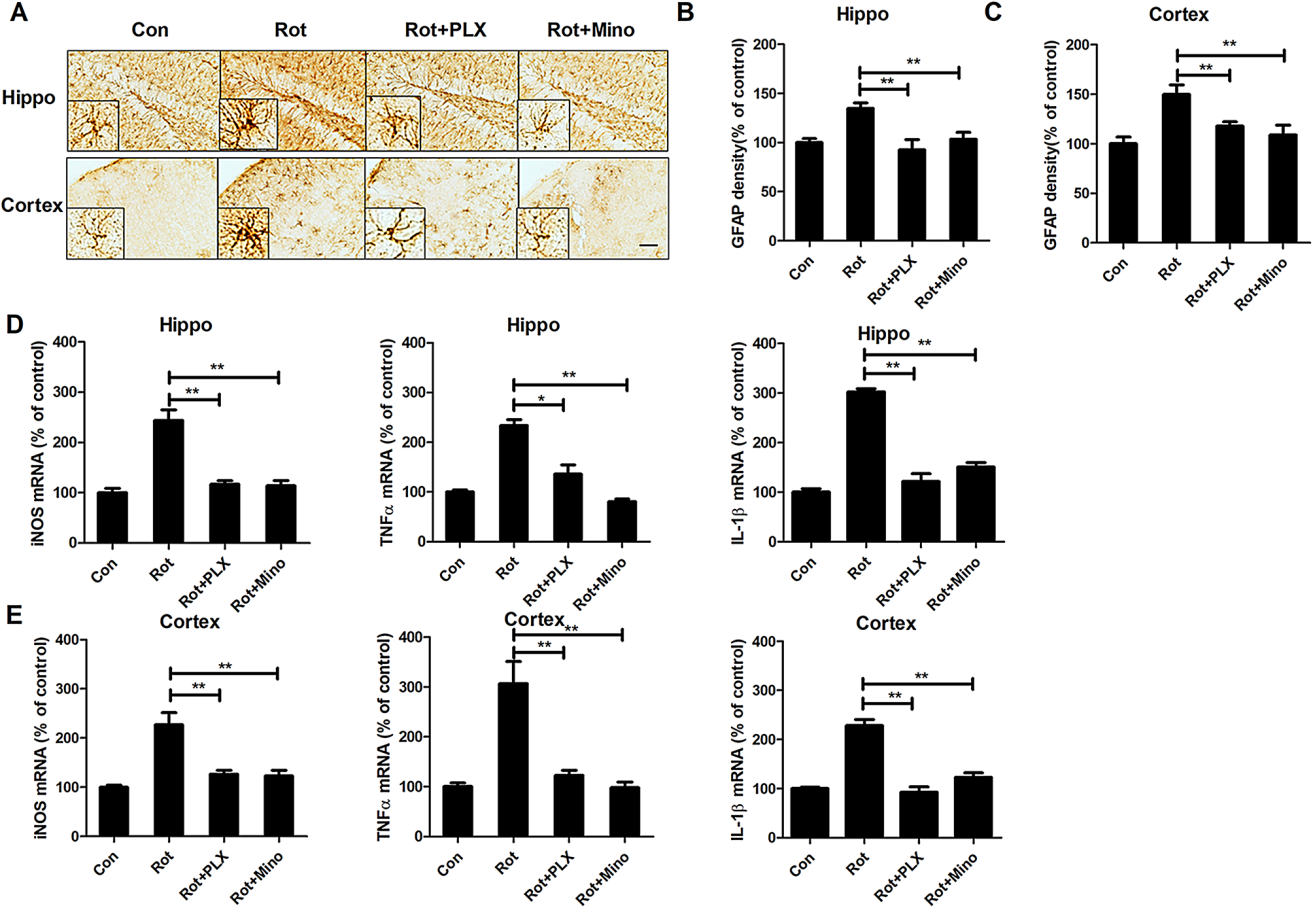
PLX3397 and minocycline attenuate rotenone-induced astroglial activation and gene expression of proinflammatory factors in mice. **a** The representative images of astroglial marker, GFAP immunostaining were shown. **b**, **c** Quantitative analysis of GFAP immunostaining density. **d**, **e** The gene expression levels of iNOS, TNFα, and IL-1β in the hippocampus (**d**) and cortex (**e**) of mice were detected. *n* = 5–6. *p < 0.05, **p < 0.01; Scale bar = 200 μm

**Correct Fig.** 6A

**Fig. 6:**
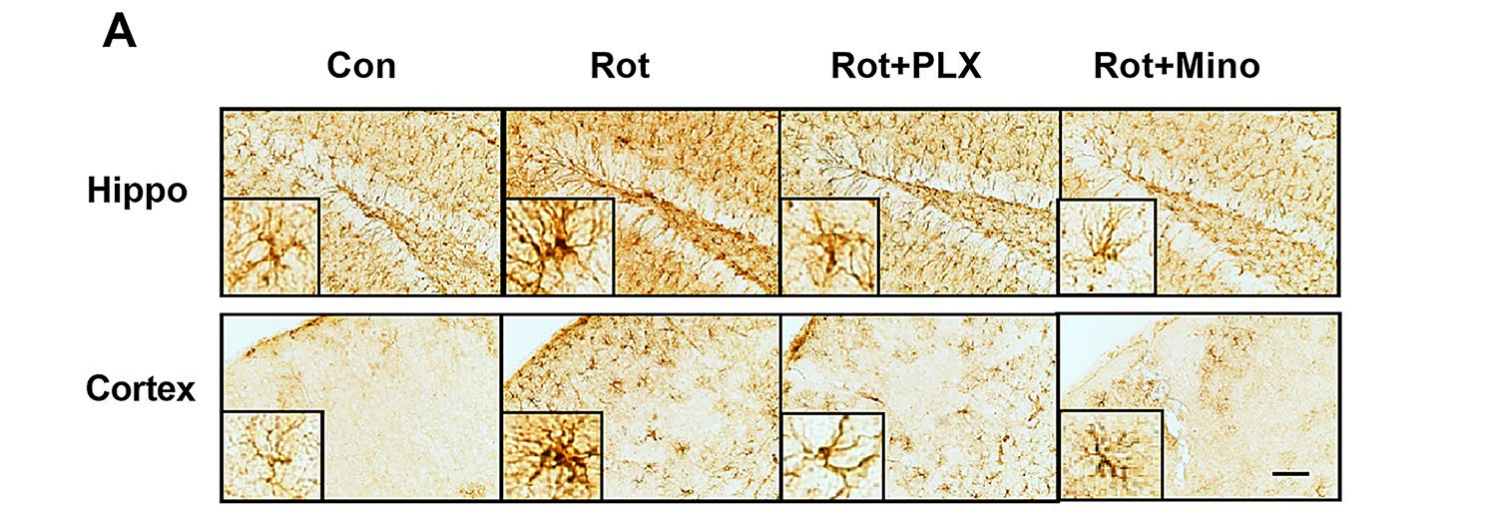
PLX3397 and minocycline attenuate rotenone-induced astroglial activation and gene expression of proinflammatory factors in mice. **a** The representative images of astroglial marker, GFAP immunostaining were shown
