# Supplementary material for: Microglial activation contributes to cognitive impairments in rotenone-induced mouse Parkinson’s disease model
Source: J Neuroinflammation. 2021 Jan 5;18:4. doi: 10.1186/s12974-020-02065-z (PMC7786472; doi:10.1186/s12974-020-02065-z)
Supplement: Supplementary file 1 — Additional file 1 Supplementary Fig 1. Rotenone dose-dependently reduces the expression of PSD-95 in CA1, CA2 and CA3 regions of mice. Quantitative analysis of PSD95 immunostaining density in CA1 (A), CA2 (B) and CA3 (C) regions of mice. ** p < 0.01. Supplementary Fig 2. The effects of PLX3397 and minocycline on microglial number and activation, respectively. (A) Mice were administered with PLX3397 (40 mg/kg/day) by gavage for 1 week and the representative images of Iba-1 immunostaining were shown. (B, C) Quantitative analysis of Iba-1+ cell number. n = 3. (D) The representative images of Iba-1 immunostaining in rotenone-treated mice with or without minocycline were shown. (E, F) Quantitative analysis of Iba-1 immunostaining density. n = 5. * * p < 0.01; Scale bar= 50 μm. Supplementary Fig 3. PLX3397 and minocycline attenuate rotenone-induced reduction of PSD95 in CA1, CA2 and CA3 regions of mice. Quantitative analysis of PSD95 immunostaining density in CA1 (A), CA2 (B) and CA3 (C) regions of mice. ** p < 0.01. Supplementary Fig 4. PLX3397 and minocycline attenuate rotenone-induced neuronal damage in mice. The representative images (40×) of PSD95 immunostaining in rotenone-treated mice with or without PLX3397 and minocycline were shown. n=5-6; Scale bar= 100 μm. Supplementary Fig 5. The effects of PLX3397 and minocycline on expession of iNOS and Arg-1 in the midbrain of rotenone-treated mice. (A) The expression levels of iNOS and arginase-1 (Arg-1) were detected in the midbrain of mice treated with rotenone with or without PLX3397 and minocycline by using Western blot and the representative blots were shown. (B) The blots was quantified. n=4; *p < 0.05, **p < 0.01. [file 12974_2020_2065_MOESM1_ESM.docx]

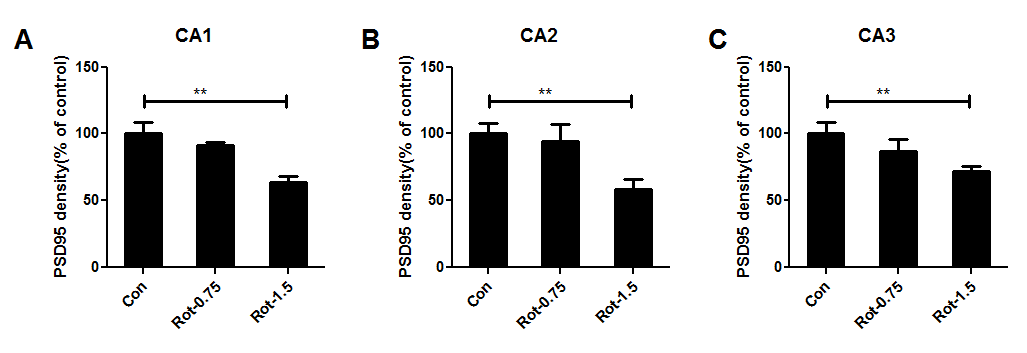


**Supplementary Fig 1. Rotenone dose-dependently reduces the expression of PSD-95 in CA1, CA2 and CA3 regions of mice.** Quantitative analysis of PSD95 immunostaining density in CA1 (A), CA2 (B) and CA3 (C) regions of mice. ** p < 0.01.


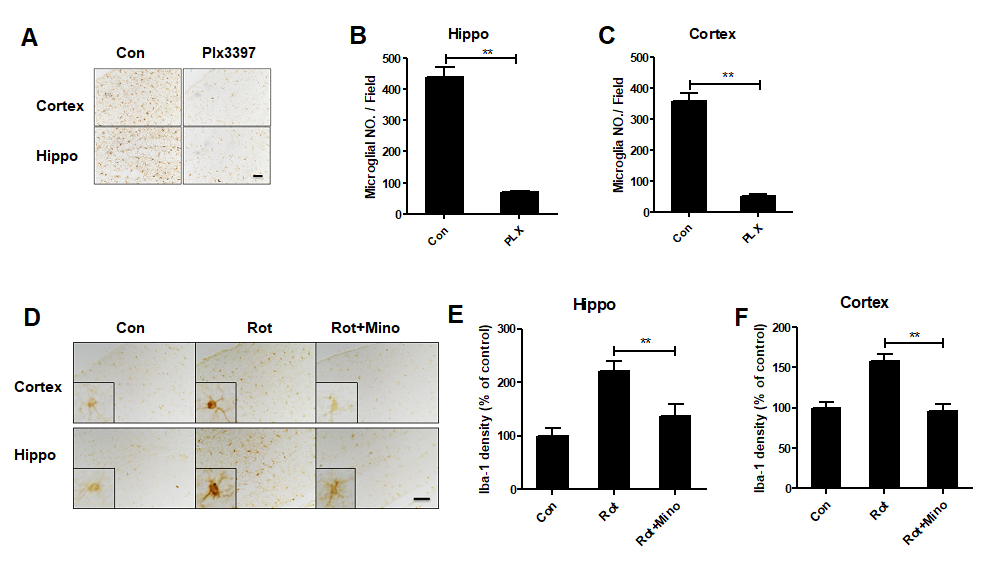


**Supplementary Fig 2. The effects of** **PLX3397 and minocycline on microglial number and activation, respectively.** (A) Mice were administered with PLX3397 (**40 mg/kg/day) by gavage** for 1 week and the representative images of Iba-1 immunostaining were shown. (B, C) Quantitative analysis of Iba-1^+^ cell number. **n = 3.** (D) The representative images of Iba-1 immunostaining in rotenone-treated mice with or without minocycline were shown. (E, F) Quantitative analysis of Iba-1 immunostaining density. **n = 5.** * * p < 0.01; Scale bar= 50 μm.


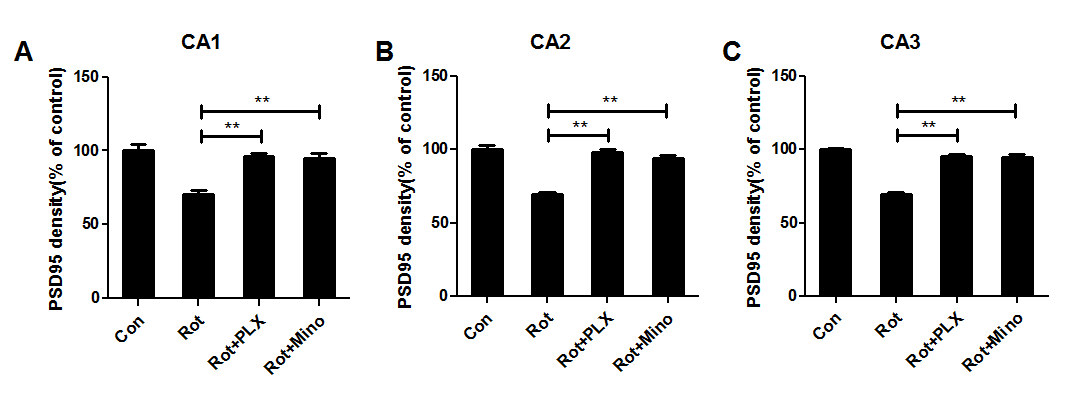


**Supplementary Fig 3. PLX3397 and minocycline attenuate rotenone-induced reduction of PSD95 in CA1, CA2 and CA3 regions of mice.** Quantitative analysis of PSD95 immunostaining density in CA1 (A), CA2 (B) and CA3 (C) regions of mice. ** p < 0.01.


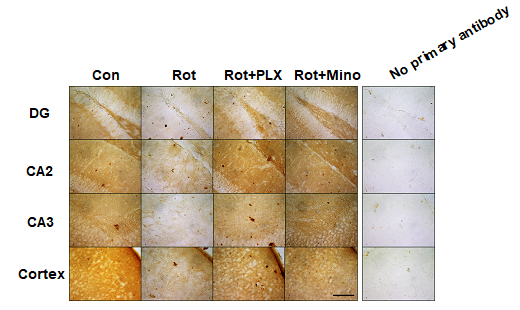


**Supplementary Fig 4. PLX3397 and minocycline attenuate rotenone-induced neuronal damage in mice.** The representative images (40×) of PSD95 immunostaining in rotenone-treated mice with or without PLX3397 and minocycline were shown. n=5-6; Scale bar= 100 μm.


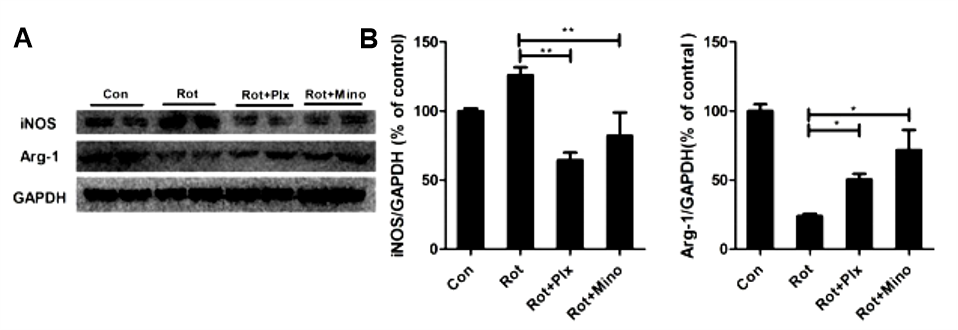


**Supplementary Fig 5.** The effects of PLX3397 and minocycline on expession of iNOS and Arg-1 in the midbrain of rotenone-treated mice. (A) The expression levels of iNOS and arginase-1 (Arg-1) were detected in the midbrain of mice treated with rotenone with or without PLX3397 and minocycline by using Western blot and the representative blots were shown. (B) The blots was quantified. n=4; *p < 0.05, **p < 0.01.
